# An item's status in semantic memory determines how it is recognized: Dissociable patterns of brain activity observed for famous and unfamiliar faces

**DOI:** 10.1016/j.neuropsychologia.2018.08.004

**Published:** 2018-10

**Authors:** Graham MacKenzie, Georgia Alexandrou, Peter J.B. Hancock, David I. Donaldson

**Affiliations:** Faculty of Natural Sciences, University of Stirling, Stirling FK9 4LA, United Kingdom.

**Keywords:** Recognition memory, Face recognition, Episodic memory, Semantic memory, Recollection, ERPs

## Abstract

Are all faces recognized in the same way, or does previous experience with a face change how it is retrieved? Previous research using human scalp-recorded Event-Related Potentials (ERPs) demonstrates that recognition memory can produce dissociable brain signals under a variety of circumstances. While many studies have reported dissociations between the putative ‘dual processes’ of familiarity and recollection, a growing number of reports demonstrate that recollection itself may be fractionated into component processes. Many recognition memory studies using lexical materials as stimuli have reported a left parietal ERP old/new effect for recollection; however, when unfamiliar faces are recollected, an anterior effect can be observed. This paper addresses two separate hypotheses concerning the functional significance of the anterior old/new effect: perceptual retrieval and semantic status. The perceptual retrieval view is that the anterior effect reflects reinstatement of perceptual information bound up in an episodic representation, while the semantic status view is that information not represented in semantic memory pre-experimentally elicits the anterior effect instead of the left parietal effect. We tested these two competing accounts by investigating recognition memory for unfamiliar faces and famous faces in two separate experiments, in which same or different pictures of studied faces were presented as test items to permit brain activity associated with retrieving face and perceptual information to be examined independently. The difference in neural activity between same and different picture hits was operationalized as a pattern of activation associated with perceptual retrieval; while the contrast between different picture hits and correct rejection of new faces was assumed to reflect face retrieval. In Experiment 1, using unfamiliar faces, the anterior old/new effect (500–700 ms) was observed for face retrieval but not for perceptual retrieval, challenging the perceptual retrieval hypothesis. In Experiment 2, using famous faces, face retrieval was associated with a left parietal effect (500–700 ms), supporting the semantic representation hypothesis. A *between-subjects* analysis comparing scalp topography across the two experiments found that the anterior effect observed for unfamiliar faces is dissociable from the left parietal effect found for famous faces. This pattern of results supports the hypothesis that an item's status in semantic memory determines how it is recognized.

## Highlights

• Famous faces are recognized more easily than unfamiliar faces.
• Left parietal ERP old/new effect is observed for famous faces.
• Anterior ERP old/new effect is observed for unfamiliar faces.
• Semantic status determines how faces are recognized.

## 1 Introduction

Is the way that you recognize somebody you have just met for the very first time the same as the way you recognize a familiar person? Some recent investigations of brain function imply that there are at least two different neural processing routes associated with face recognition (Galli and Otten, 2011, Nie et al., 2014). Here we describe two separate recognition memory experiments for faces investigating patterns of neural activity with Event-Related Potentials (ERPs), which seek to determine why recognizing somebody you have just met may differ from recognizing a familiar person. First, however, we briefly introduce recognition memory and how experiments using ERPs have been instrumental in highlighting the role that semantic memory might play in determining how we recognize faces.

Episodic memory is the system that allows us to re-experience the past in the present moment, and along with future thinking supports the human capacity for ‘mental time travel’ (Tulving, 1985a). In the laboratory, episodic memory is typically investigated using one of two types of test: recall or recognition. While the free recall of information about past experiences arguably better captures the phenomenological experience of remembering, recognition memory tests are popular with cognitive neuroscientists because they allow for precise control of experimental parameters. In the typical recognition memory experiment participants are presented with a list of items to study and after some delay are presented with a test list containing a mix of studied (old) and non-studied (new) items. The participant's task is to accurately categorize test items as either ‘old’ or ‘new’. Theoretical models attempting to account for recognition memory task behaviour can be distinguished by the number of retrieval processes that are proposed to support performance. While some experts argue that one single retrieval process supports both ‘weak’ and ‘strong’ item recognition (e.g., Dunn, 2004; Squire et al., 2007), dual process models describe two dissociable retrieval processes called ‘familiarity’ and ‘recollection’ (Mandler, 1980, Jacoby and Dallas, 1981, Yonelinas, 1994). Common to most dual process models is the view that familiarity provides information about an item's memory strength, whereas recollection provides information concerning the context in which an item was encoded. When applied to recognition memory for a person, for example, a sense of pure familiarity is experienced when a person is confidently recognized but cannot be placed. By contrast, recollection would involve both recognition of the person and concomitant retrieval of associated information, such as where or when the person was previously encountered.

Empirical evidence supporting dual process models comes from multiple fields, including experimental cognitive psychology (Topolinski, 2012), neuropsychology (Aggleton et al., 2005, Bowles et al., 2010) and functional neuroimaging (Vilberg and Rugg, 2007). Particularly compelling evidence comes from recognition memory studies using ERPs, which provide a measure of neural processing derived from electrical fields detected on the scalp. Stimulus-locked ERP studies identify a pattern of neural activity called the ‘old/new effect’, which is a voltage difference between correctly identified old and new test items, typically becoming apparent approximately 300 ms post-stimulus onset. Crucially, old/new effects can be observed at more than one scalp location or during more than one latency period relative to stimulus-onset, and effects with dissociable functional, topographic and temporal characteristics have been linked with familiarity and recollection (Curran, 2000, Curran and Cleary, 2003, Rugg et al., 1998). Specifically, a large number of studies have reported an early frontal old/new effect (or *FN400*), maximal during the 300–500 ms latency period, reflecting neural processing linked to familiarity (Kamp et al., 2016, Strozak et al., 2016) and a left parietal old/new effect (500–700 ms) associated with processes related to recollection (see Rugg and Curran, 2007; Bergstrom et al., 2016).^1^ However, the generality of this typical pattern of old/new effects has been questioned by several more recent studies demonstrating that recollection can sometimes elicit an alternative ERP old/new effect with an anterior scalp distribution (Galli and Otten, 2011, MacKenzie and Donaldson, 2007), which can be dissociated from both the left parietal recollection effect and the early mid-frontal familiarity effect (MacKenzie and Donaldson, 2009).

The first study reporting the alternative anterior recollection ERP old/new effect by MacKenzie and Donaldson (2007) was designed to investigate whether unfamiliar faces could be used to identify a pure familiarity signal, since unfamiliar faces should not be contaminated with previous episodic memories. In the study phases, each unfamiliar face was paired with a unique first name, which was spoken in a male or female voice to match the gender of the face. In the test phases, old faces were re-presented without their associated names, intermixed with new faces. The task required participants to make an initial old/new decision to each face, with secondary *name*/*other specifics*/*no specifics* decisions for all faces that were recognized. Inspired by Yovel and Paller (2004), on whose design the experiment was based, the *name* and *other specifics* responses were intended to capture recollection, whereas the *no specifics* response was intended to capture familiarity. In their study MacKenzie and Donaldson (2007) observed an ERP old/new effect with an anterior distribution from approximately 400 to 800 ms for faces that were recollected, and this anterior effect was bigger when names were retrieved than when other specific information was retrieved. Importantly, this recollection effect was topographically dissociated from a posterior old/new effect observed for familiarity. The anterior-posterior topographic dissociation is consistent with dual process models because it implies that the ERP effects observed for recollection and familiarity are due to activation of at least partially non-overlapping neural populations. In addition, the discovery of an anterior old/new effect for recollection of faces was noteworthy because there was no clear evidence of the left parietal effect typically associated with recollection, suggesting a face-specific brain signal.

Three hypotheses have now been advanced to account for the atypical recollection ERP effects observed for unfamiliar faces. First, MacKenzie and Donaldson's (2007) ‘face specificity’ hypothesis contended that faces might be recollected in a different way from other types of information. This hypothesis received support in a subsequent study using Tulving (1985b) Remember/Know procedure in which remembered unfamiliar faces were associated with an anterior effect and remembered names were associated with a left parietal effect (MacKenzie and Donaldson, 2009). An anterior effect was also observed for unfamiliar faces by Yick and Wilding (2008) – although in this case no process estimation procedure was used and therefore the effect could not be linked with either familiarity or recollection. However, more recently the face specificity hypothesis was fundamentally challenged by Galli and Otten (2011), who observed an equivalent anterior old/new effect for recollection of high quality photographic representations of common objects, as well as for recollection of unfamiliar faces. This observation led Galli and Otten to speculate that the anterior recollection effect reflects the retrieval of perceptual information from past episodes, and that the material-specificity of recollection was chiefly concerned with the distinction between verbal and pictorial information. To date, Galli and Otten's ‘perceptual retrieval’ hypothesis has not been investigated further, hence one aim of the current study is to test this account against a third alternative: the ‘status in semantic memory’ view.

Based on previous findings that it is easier to remember items with long term memory representations than novel items (Reder et al., 2006, Reder et al., 2013), Nie et al. (2014) advanced the semantic status interpretation of the anterior ERP old/new effect in a paper reporting an elegant item recognition experiment. The authors compared ERP old/new effects for words and faces that were either represented in semantic memory pre-experimentally or not (words, pseudowords, famous faces, unfamiliar faces). Replicating the results of MacKenzie and Donaldson (2009), Nie et al. observed an anterior effect for unfamiliar faces, which contrasted with a left parietal effect for words. Crucially, the effect observed for famous faces resembled the left parietal effect more than it resembled the effect for unfamiliar faces, consistent with the idea that the status of a stimulus in semantic memory determines how the information is retrieved. Given the scalp distribution and timing of the anterior effect, one implication of this ‘status in semantic memory’ view is that stimuli that do not have pre-existing representations in semantic memory may be recollected in a different way from stimuli that are associated with specific semantic representations, such as words, which are typically associated with a left parietal effect. Here, across two experiments, we further explore the functional significance of the anterior ERP old/new effect, testing the perceptual retrieval and status in semantic memory accounts.

The starting point for the current pair of experiments stems from the literature on face processing, rather than episodic memory *per se.* Hancock et al. (2000) argued that the only way to truly demonstrate face recognition is where different pictures of studied faces are recognized. This constraint matters because recognition of the same picture that was studied could be supported by retrieval of perceptual information from the picture rather than retrieval of the face itself. Based on this reasoning, one way to isolate brain activity associated with successful retrieval of perceptual information that is bound up in an episodic representation is to contrast ERP waveforms for same and different pictures of studied faces. In principle, this contrast should reveal a pattern of brain activity for the retrieval of perceptual information from the episodic representation; and if an anterior effect is observed then the perceptual retrieval hypothesis will be supported. Moreover, the contrast between different hit and new ERPs should identify brain activity associated with face retrieval. Thus, by separating test phase ERPs into same and different picture hits, along with correct rejections of new faces, we can examine patterns of neural activity related to both perceptual retrieval (same hit vs. different hit) and face retrieval (different hit vs. new).

Below, we present a pair of experiments designed to investigate the functional significance of the anterior old/new effect (500–700 ms). Our aim is to examine two competing accounts: perceptual retrieval and semantic status. The first study uses unfamiliar faces, whereas the second study uses famous faces. The key difference between these two sets of stimuli is their status in semantic memory.

## 2 Experiment 1

Experiment 1 is an item recognition task using unfamiliar faces as stimuli. Retrieval cues were manipulated, with half of the old faces tested using the same picture that was encoded and half tested with a different picture. Consistent with the theoretical accounts outlined above, we hypothesised that if the anterior ERP old/new effect reflects perceptual retrieval, then the same/different hit ERP comparison should have a frontal maximum from 500 to 700 ms. By contrast, if the anterior effect reflects semantic status, then the different hit/new ERP contrast will have a frontal maximum, because unfamiliar faces are not represented in semantic memory pre-experimentally and are therefore more likely to produce an anterior effect than a left parietal effect.

### 2.1 Experiment 1 materials and methods

Twenty-four right-handed participants (13 female) with a mean age of 24 years (range: 18–28) and self-report of no neurological problems took part in the study. Participants were recruited from the student population at the University of Stirling, and were compensated at a rate of £ 7.50 per hour. Participants provided written consent after reading the task instructions and information about the EEG recording procedure. All experimental methods and procedures were approved by the University of Stirling Psychology Ethics Committee.

Experimental stimuli consisted of 2 different photographs of each of 400 unfamiliar faces. The stimuli used for the study were taken from The Glasgow Unfamiliar Face Database (Burton et al., 2010), the NBU Face Database (New Bulgarian University Face Database, 2006) and from the Psychological Image Collection at Stirling (PICS; pics.stir.ac.uk). The colour photographs were cropped to exclude background information but the external features of the faces such as hairline, etc. remained untouched. Faces were centred and set against a black background. An equal number of male and female faces was used. Stimuli were shown on a 17″ colour LCD monitor. The size of the image boundary was 350 pixels wide by approximately 450 pixels high, providing a horizontal visual angle of 15.9° and a vertical visual angle of 18.2°.

The item recognition memory task was sub-divided into 10 study-test blocks. Each block contained 20 faces presented at study, followed at test by 10 same-picture, 10 different-picture and 20 new faces. The stimuli were counterbalanced across participants so that each face had an equal chance of serving as an old or new item, and so that each old face appeared in the same and different conditions an equal number of times. Each block was randomised so that the selection of faces would counter against any order of presentation effects. Each study trial began with a white fixation cross (+) presented in the centre of the screen against a black background for 1000 ms, followed immediately by a face stimulus presented centrally for 2000 ms, which was in turn followed by a blank black screen for 1000 ms. Each test trial began with a central white fixation cross for 1000 ms, followed immediately by a face for 2000 ms, and then by a blank screen. Participants were asked to make an old/new response to each face, and the response was registered either during the presentation of the face or during the blank screen that followed. Participants made responses with fingers or thumbs from both hands by using a button box. The allocation of left or right buttons to old and new responses was counterbalanced across participants.

During testing, EEG was recorded from 64 scalp electrodes embedded in a cap and referenced to an electrode positioned between the CZ and CPZ sites. Electrode positions were based on the extended 10–20 System (Jasper, 1958). Two additional EEG electrodes were placed on the right and left mastoids. Bipolar electro-oculogram (EOG) was recorded from electrodes placed above and below the left eye (VEOG) and on each temple (HEOG). Impedances were kept below 5 kΩ. Scan 4.2 was used to record the data, which were filtered between 40 and 0.01 Hz during acquisition. Blink artefacts were removed using a regression procedure (Semlitsch et al., 1986). Trials were segmented into 1100 ms epochs beginning 100 ms before the onset of stimuli. Trials were baseline corrected to the average of the pre-stimulus interval, re-referenced offline to the average of the two mastoid channels, and smoothed over a 5-point kernel. Trials were excluded if drift (defined as the difference between the first and last data points) exceeded ± 75 µV or if activity anywhere in an epoch exceeded ± 100 µV. To ensure a good signal-to-noise ratio, a minimum of 16 artefact-free trials per condition was set as a criterion before including a participant's data in grand-average ERPs.

**Fig. 1:**
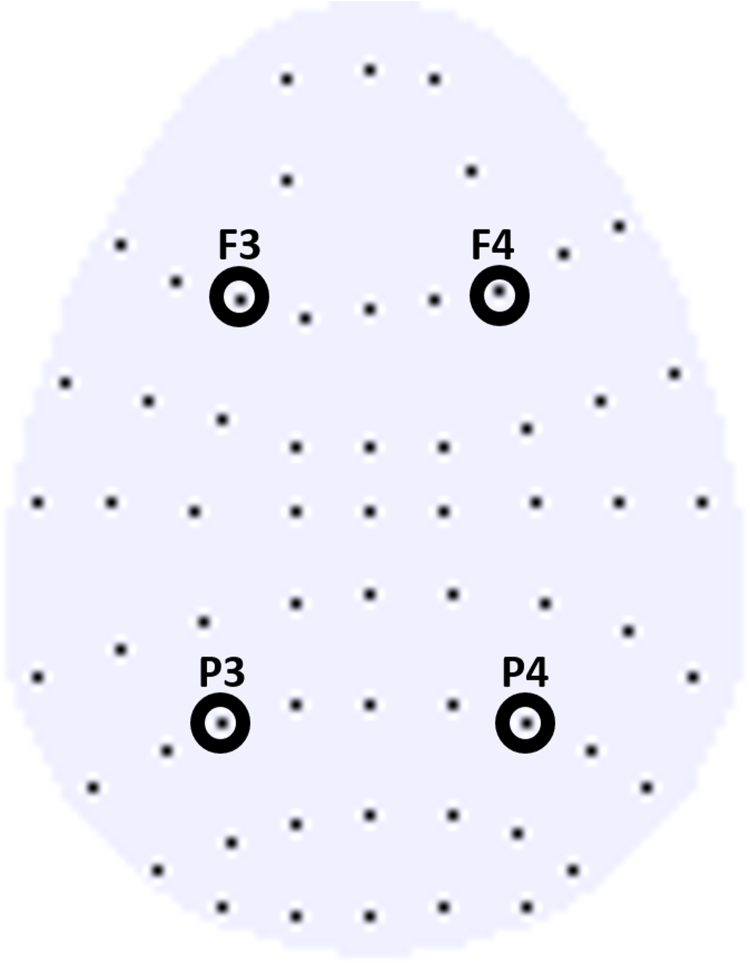
Electrode montage. The scalp map represents a view of the electrode montage, which is based on the 10-20 system (Jasper, 1958). The front of the head is at the top and the left hemisphere is on the left. Each black dot represents an electrode, and the electrodes used for analysis are highlighted with black circles and labelled. For the ANOVA, the four electrodes used for analysis are grouped into two factors: location (frontal vs. parietal) and hemisphere (left vs. right).

Waveforms were quantified by computing the average voltage in consecutive latency periods from 300 to 500 ms and from 500 to 700 ms, reflecting *a priori* definitions of the time windows in which the neural correlates of familiarity and recollection are typically observed. Our analysis strategy involved two stages. First, we carried out omnibus analyses to characterise the broad differences between the waveforms and so that we could apply one common ANOVA model to all analyses, including the unfamiliar and famous face effects across experiments. Second, we performed focused analyses that target the specific hypotheses outlined in the introduction. The initial evaluation employed repeated measures analyses of variance (ANOVA), initially with factors of condition (same/different/new), location (frontal/parietal), and hemisphere (left/right). Electrodes used for analysis were: F3, F4, P3, and P4 (see Fig. 1). Only *p < .05* values were considered significant, and only the effects and interactions involving the critical old/new factor are reported. Any significant interactions were followed up by subsidiary analyses to help interpret higher-level effects. In the second analysis phase, targeted analyses of planned contrasts were performed to assess the two different hypotheses concerning the functional significance of the anterior old/new effect that are the subject of this paper. These secondary analyses used a repeated-measures ANOVA with factors of condition (same/different or different/new) and hemisphere (left/right) and were performed on data from the frontal and parietal locations separately.

### 2.2 Experiment 1 behavioural results

**Fig. 2:**
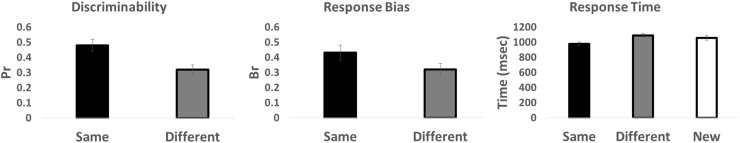
Unfamiliar face task performance. The panel on the left shows better accuracy for same than different pictures. The middle panel shows a more liberal response bias for same than different pictures. The panel on the right shows faster response times for recognising same pictures than recognising different pictures or rejecting new faces.

Fig. 2 illustrates memory performance for same and different pictures, as indexed by the discriminability index *Pr* (*Pr = Hit-FA*; Snodgrass and Corwin, 1988). Participants were clearly able to discriminate old from new pictures in both cases, but exhibited better memory for same (0.48, s.d. = 0.20) than different (0.32, s.d. = 0.15) pictures. A paired-samples *t*-test revealed a statistically reliable difference in discriminability for same and different pictures *(∆*$\overset{®}{x}$ *= 0.16 ± 0.04, t(23) = 9.0, p < .001, d = 1.84*). These data reflect variation in the proportion of old responses at test, with mean hit rates for same ($\overset{®}{x}$ *=* 0.7, s.d. = 0.17) and different ($\overset{®}{x}$ *=* 0.54, s.d. = 0.16), compared to the false alarm rate to new items ($\overset{®}{x}$ *=* 0.22, s.d. = 0.15). Fig. 1 also shows response bias (*Br = FA/(1-Pr*); Snodgrass and Corwin, 1988) for same ($\overset{®}{x}$ *=* 0.43, s.d. = 0.23) and different ($\overset{®}{x}$ *=* 0.32, s.d. = 0.19) pictures. Values of *Br* between 0 and 0.5 indicate a conservative response bias, which can be observed for both types of stimuli. A paired-samples *t*-test revealed a reliable difference in response bias between same and different pictures *(∆*$\overset{®}{x}$ *= 0.11 ± 0.05, t(23) = 5.09, p < .001, d = 1.04*). Overall, the performance data show that same pictures were recognized more often and with a more liberal response bias than different pictures.

Fig. 2 also shows response times (RTs) for correct responses to same, different and new pictures. RTs were fastest for recognition of same pictures, while RTs for different pictures and new faces were similar. A one-way repeated measures ANOVA revealed a significant difference in mean RT between the conditions [*F (2,44) = 7.70, p = .001, η*_*p*_^*2*^ *= 0.26*]. Bonferroni-corrected pair-wise comparisons revealed that hits were significantly faster for same than different pictures (*∆*$\overset{®}{x}$ = 114 ± 82, d = 0.74), but that no reliable differences were observed between same and new faces (*∆*$\overset{®}{x}$ = 74 ± 87, d = 0.45) or between different and new faces (*∆*$\overset{®}{x}$ = 40 ± 57, d = 0.37). Taken together, therefore, the behavioural data suggest that same pictures are recognized more accurately and faster than different pictures.

### 2.3 Experiment 1 electrophysiology

**Fig. 3:**
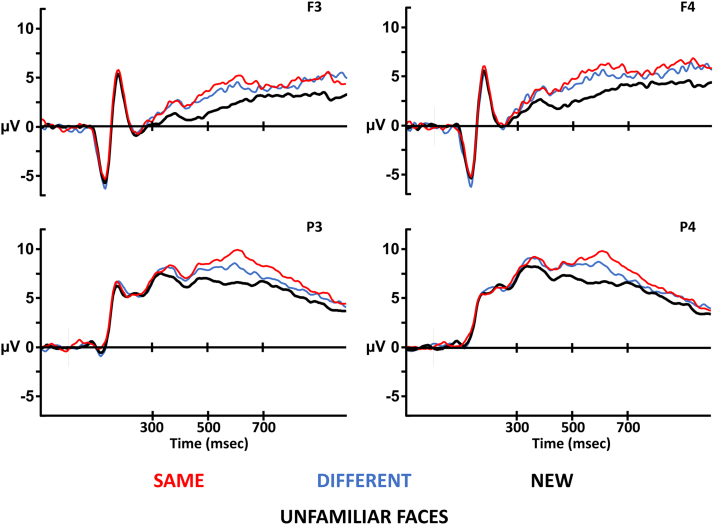
Unfamiliar face Event-Related Potentials. Waveforms for all three experimental conditions are plotted at the electrodes used for analysis. The electrodes at the top (F3/F4) come from the frontal location, and the electrodes at the bottom (P3/P4) come from the parietal location. The electrodes on the left (F3/P3) come from the left hemisphere, and the electrodes on the right (F4/P4) come from the right hemisphere. At each electrode, the x-axis depicts time (in milliseconds) relative to test stimulus onset, and the y-axis depicts amplitude (microvolts). At all electrodes, the waveform for new faces is more negative-going than the waveforms for same and different pictures from approximately 300 msec. During the critical 500–700 msec latency period, the difference between same and different waveforms reflecting perceptual retrieval is bigger at the parietal location than at the frontal location.

Grand averages were formed for correct responses only for the same, different and new conditions and the average number of trials in these conditions was 55, 43 and 126, respectively. Fig. 3 shows grand-average Event-Related Potential (ERP) waveforms for correctly identified same, different and new faces at the left- and right-hemisphere (3/4), frontal and parietal (F/P) electrodes used for analysis. The waveforms are generally more positive-going for same than different pictures, and the difference is most apparent at the parietal electrodes during the 500–700 ms latency period. By contrast, the waveform for different pictures appears to be larger than the waveform for new faces at the frontal location throughout the epoch.

#### 2.3.1 Omnibus analysis

From 300 to 500 ms, the analysis revealed a main effect of condition, *F(2,46) = 5.29, p = .009, η*_*p*_^*2*^ *= 0.19*. Bonferroni-corrected pairwise comparisons identified differences between the same and new waveforms ($\overset{®}{x}$*= 0.89 ± 0.81 μV, p = .028, d = 0.58*) and between the different and new waveforms ($\overset{®}{x}$ *= 0.86 ± 0.79 μV, p = .030, d = 0.57*); however, the difference between the same and different waveforms was not reliable ($\overset{®}{x}$ *= 0.03 ± 0.80 μV, p > .999, d = 0.02*). The main effect therefore reflects a more positive-going waveform for both same and different pictures with respect to new faces, with no difference between the same and different pictures (i.e., same = different > new). Importantly, the main effect of condition is qualified by a significant interaction between condition and location, *F(2,46) = 3.78, p = .030, η*_*p*_^*2*^ *= 0.14*, which is due to a main effect of condition being present at the frontal location, *F(2,46) = 7.47, p = .002, η*_*p*_^*2*^ *= 0.24*, but not at the parietal location, *F(2,46) = 1.40, p = .258, η*_*p*_^*2*^ *= 0.06*.

From 500 to 700 ms, the analysis identified a main effect of condition, *F(2,46) = 16.21, p < .001, η*_*p*_^*2*^ *= 0.41*. Bonferroni-corrected pairwise comparisons identified differences between the same and new waveforms ($\overset{®}{x}$ *= 2.00 ± 0.98 μV, p < .001, d = 1.07*) and between the different and new waveforms ($\overset{®}{x}$ *= 1.27 ± 0.95 μV, p = .006, d = 0.70*); however, the same/different comparison was not reliable ($\overset{®}{x}$ *= 0.73 ± 0.81 μV, p > .088, d = 0.47*). As in the earlier time window, the main effect reflects a more positive-going waveform for both same and different pictures with respect to new faces, with no difference between the same and different pictures (i.e., same = different > new).

#### 2.3.2 Perceptual retrieval

**Fig. 4:**
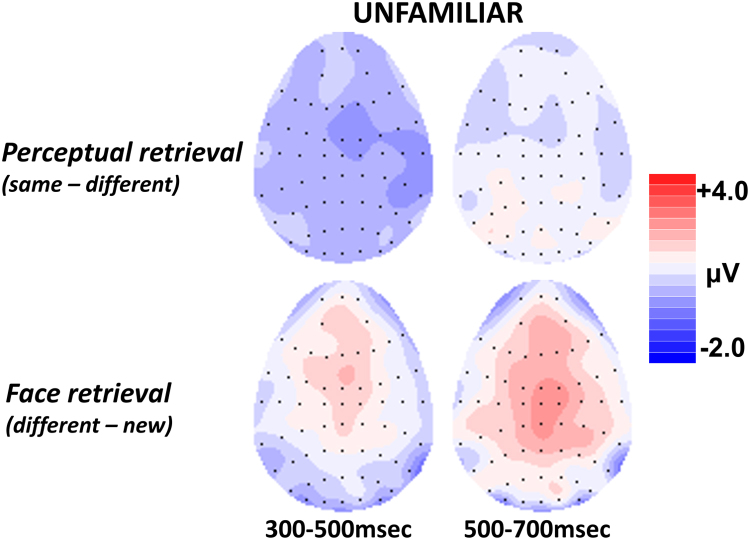
Topographic maps showing the distribution of unfamiliar face ERP effects. The different hit waveform has been subtracted from the same hit waveform to show the pattern of neural activity related to picture retrieval, and the new waveform has been subtracted from the different hit waveform to show neural activity associated with face retrieval. Scalp maps show the average neural activity during each latency period, with the front of the head at the top. Each dot represents an electrode where the size of the difference between waveforms is known; the size of the effect is interpolated between electrodes to estimate the overall scalp distribution. Red colours represent areas where the difference between waveforms is most positive. The scale bar represents the size of the ERP effects in microvolts. The pattern of neural activity observed for picture retrieval is posteriorly distributed during the critical 500–700 msec latency period associated with ERP signals associated with recollection. By contrast, neural activity observed for face retrieval extends over frontal scalp.

To assess the perceptual retrieval hypothesis, a targeted analysis of the same and different hit waveforms at the frontal location was performed, examining whether an anterior effect was observed. From 500 to 700 ms, the main effect of condition was not reliable at frontal electrodes, *F(1,23) = 2.54, p = .125, η*_*p*_^*2*^ *= 0.10*. To assess whether the lack of an effect at the frontal location might stem from a lack of power, the parietal electrodes were additionally analysed and a reliable main effect was observed, *F(1,23) = 8.95, p = .007, η*_*p*_^*2*^ *= 0.28*. Thus, whilst perceptual retrieval elicited significant differences in neural activity over posterior scalp, no evidence was found for the anterior effect predicted by the perceptual retrieval hypothesis. Fig. 4 shows the scalp topography of the perceptual retrieval and face retrieval effects.

#### 2.3.3 Face retrieval

To assess the status in semantic memory hypothesis, the different and new waveforms were compared directly to see if an old/new effect was present at the frontal location during the latency period in which the anterior effect is typically observed. In the 500–700 ms latency period the main effect of condition was reliable at frontal electrodes, *F(1,23) = 12.18, p = .002, η*_*p*_^*2*^ *= 0.35.* As for perceptual retrieval, we also carried out additional analysis focused on data from the parietal location. Comparison of the different and new waveforms revealed a reliable main effect of condition at parietal electrodes, *F(1,23) = 8.95, p = .007, η*_*p*_^*2*^ *= 0.28*. Examination of the magnitude of the effects at frontal and parietal locations reveals that the effect is better characterised as an effect with a frontal maximum: 1.42 μV at the frontal location vs. 1.12 μV at the parietal location.

### 2.4 Experiment 1 results summary

Experiment 1 used unfamiliar faces to examine the perceptual retrieval hypothesis of the functional significance of the anterior ERP old/new effect. Galli and Otten (2011) advanced the hypothesis that the anterior effect reflects processes linked with the reinstatement of perceptual processes involved in the encoding of an episode. Experiment 1 contrasted ERPs for same and different picture hits to reveal neural activity associated with perceptual retrieval. If the functional significance of the anterior effect reflects perceptual retrieval, then the contrast between same and different hit waveforms should have a frontal maximum. However, only a weak posterior effect was observed for the same/different face contrast, which fails to support the perceptual retrieval hypothesis. However, an anterior old/new effect was observed in the contrast between different hits and new faces, designed to reflect face retrieval. This finding provides partial support for a competing account of the functional significance of the anterior effect: namely, the status in semantic memory hypothesis.

## 3 Experiment 2

A second experiment was performed to assess whether the left parietal old/new effect is associated with familiar face recognition, as predicted by the status in semantic memory theory (Nie et al., 2014). The design of the experiment is the exact same as experiment 1, with the only change being the substitution of famous faces for the unfamiliar face stimuli. Thus, across the two experiments the status of the stimuli in semantic memory will be manipulated. If it is indeed an item's status in semantic memory that determines whether or not the anterior effect is observed, then famous faces should be associated with an ERP old/new effect that differs from the anterior effect observed for unfamiliar faces in experiment 1. More specifically, the status in semantic memory theory predicts that a left parietal effect will be observed for famous faces.

### 3.1 Experiment 2 materials and methods

Twenty-four right-handed participants were tested but data from one participant were discarded for failure to provide enough trials to form grand-average ERPs for all three experimental conditions. Accordingly, data from 23 participants (13 female) with a mean age of 20 years (range: 18–26) and self-report of no neurological problems are reported here. Participants were recruited from the student population at the University of Stirling, and were compensated at a rate of £7.50 per hour. Participants provided written consent after reading through the task instructions and information about the EEG recording procedure. All experimental methods and procedures were approved by the University of Stirling Psychology Ethics Committee.

Experimental materials consisted of 2 different colour photographs of each of 400 famous faces. The identities were selected in the hope that their faces would be readily recognizable by a cohort of young adults studying at a Scottish university. All photographs were sourced from the internet, and cropped and resized according to the parameters reported above for experiment 1.

The design of the experiment was as described above for experiment 1, except for the stimuli and that after completing the task and removal of the electrode cap, participants performed a final identity check task to gauge whether or not the famous face stimuli used in the experiment were familiar to them. This task was performed on a computer, and a different set of photographs from the ones used in the main task were employed. Stimuli were presented in a random order and remained on screen until the participant indicated by button process whether they were familiar with the person or not. Allocation of left- and right-hand buttons to familiar/unfamiliar response options was counterbalanced across participants. Familiarity with the identities was defined as face recognition rather than person identification *per se*. Faces of famous people flagged in this identity check task as unfamiliar were excluded from both the behavioural and ERP data for the main experiment.

### 3.2 Experiment 2 behavioural results

**Fig. 5:**
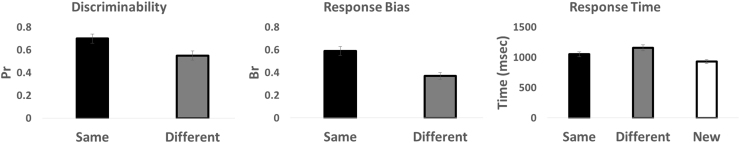
Famous face task performance. The panel on the left shows better accuracy for same than different pictures. The middle panel shows a more liberal response bias for same than different pictures. The panel on the right shows faster response times for recognising same pictures than recognising different pictures.

Participants were familiar with a mean of 64% (s.d. = 19%) of the identities used in the recognition memory task. Faces with whom participants were unfamiliar have been excluded from the following data and analyses. Fig. 5 illustrates memory performance for same and different pictures, as indexed by the discriminability index *Pr*. Participants were clearly able to discriminate old from new pictures in both cases, but exhibited better performance for same ($\overset{®}{x}$ *=* 0.70, s.d. = 0.19) than different ($\overset{®}{x}$ *=* 0.55, s.d. = 0.18) pictures. A paired-samples *t*-test carried out on the *Pr* data revealed a statistically reliable difference in memory for same and different pictures *(∆*$\overset{®}{x}$ *= 0.14 ± 0.03, t(22) = 11.74, p < .001, d = 2.45*). These data reflect differences in the proportion of old responses at test, with mean hit rates for same ($\overset{®}{x}$ *=* 0.87, s.d. = 0.11) and different ($\overset{®}{x}$ *=* 0.73, s.d. = 0.11), compared to the false alarm rate to new items ($\overset{®}{x}$ *=* 0.17, s.d. = 0.13). Fig. 4 also shows response bias (*Br*) for same ($\overset{®}{x}$ *=* 0.59, s.d. = 0.20) and different ($\overset{®}{x}$ *=* 0.37, s.d. = 0.14) pictures. A liberal bias is associated with same pictures, whereas a conservative bias can be observed for different pictures. A paired-samples *t*-test carried out on the *Br* data revealed a reliable difference in response bias between same and different pictures *(∆*$\overset{®}{x}$*= 0.23 ± 0.06, t(22) = 7.86, p < .001, d = 1.64*). Overall, the performance data show that same pictures were recognized more often and with a more liberal bias than different pictures.

Fig. 5 also shows response times (RTs) for correct responses to same, different and new pictures. RTs were fastest for correct recognition of same pictures, and fastest for new faces. A one-way repeated measures ANOVA revealed a significant difference in mean RT between the conditions [*F (2,44) = 56.35, p < .001, η*_*p*_^*2*^ *= 0.72*]. Bonferroni-corrected pair-wise comparisons revealed that same picture hits were significantly faster than different picture hits (*∆*$\overset{®}{x}$= 119 ± 44, d = 1.46), but slower than new faces (*∆*$\overset{®}{x}$ = 229 ± 66, d = 1.82), and also that different picture hits were significantly slower than new faces (*∆*$\overset{®}{x}$= 110 ± 53, d = 1.11). Taken together, therefore, the behavioural data suggest that same pictures are recognized more accurately and faster than different pictures.

### 3.3 Experiment 2 electrophysiology

**Fig. 6:**
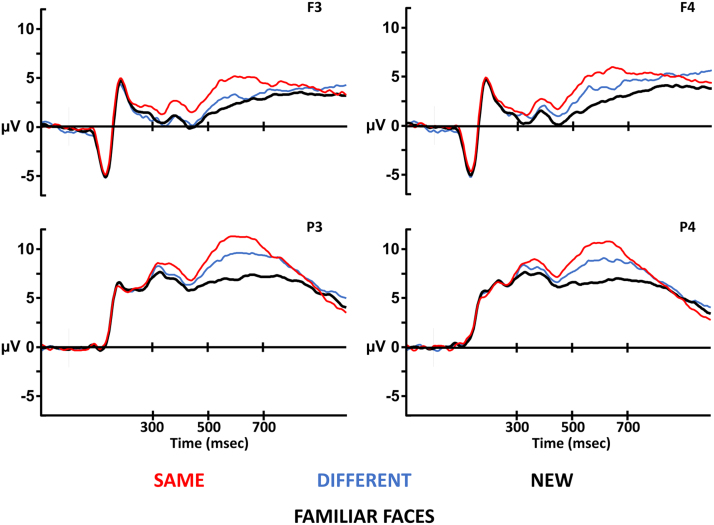
Famous face Event-Related Potentials. Waveforms for all three experimental conditions are plotted at the electrodes used for analysis. The electrodes at the top (F3/F4) come from the frontal location, and the electrodes at the bottom (P3/P4) come from the parietal location. The electrodes on the left (F3/P3) come from the left hemisphere, and the electrodes on the right (F4/P4) come from the right hemisphere. At each electrode, the x-axis depicts time (in milliseconds) relative to test stimulus onset, and the y-axis depicts amplitude (microvolts). At all electrodes, the waveform for new faces is more negative-going than the waveforms for same and different pictures from approximately 300 msec. At frontal electrodes, the waveform for same pictures is more positive-going than the waveform for different pictures before 300 msec. During the critical 500–700 msec latency period, the difference between different and new waveforms reflecting face recognition is bigger at the parietal location than at the frontal location.

Grand averages were formed for correct responses only for the same, different and new conditions for which the average number of trials was 52, 43 and 92, respectively. Fig. 6 shows grand-average ERP waveforms for all three conditions at the frontal and parietal electrodes used for analysis. The waveform for same pictures is more positive-going than the waveform for different pictures from approximately 200 ms at frontal electrodes; the difference between the waveforms appears to be more pronounced at the frontal electrodes during the 500–700 ms latency period. The waveform for different pictures of familiar faces that were correctly recognized diverges from the waveform for correctly rejected new familiar faces around 300 ms at frontal electrodes; this divergence appears to be more pronounced at the left parietal electrode from 500 m to 700 ms.

Waveforms for all three conditions were quantified as the mean amplitude in two consecutive 200 ms latency periods from 300 ms. Data were submitted to ANOVA with factors of condition (same/different/new), location (frontal/parietal) and hemisphere (left/right). Only main effects and interactions involving the condition factor are reported.

#### 3.3.1 Omnibus analysis

From 300 to 500 ms, the analysis revealed a main effect of condition, *F(2,44) = 9.81, p < .001, η*_*p*_^*2*^ *= 0.31*, and an interaction between condition, location, and hemisphere, *F(1.41,31.05) = 5.64, p = .015, η*_*p*_^*2*^ *= 0.20*. Bonferroni-corrected pair-wise comparisons found differences between the waveforms for same and new ($\overset{®}{x}$ *= 1.59 ± 1.03μV, p = .002*) and the waveforms for different and new ($\overset{®}{x}$ *= 0.98 ± 0.81μV, p = .014I*) but not between the same and different waveforms ($\overset{®}{x}$ *= 0.61 ± 0.96 μV, p = .352*). The three-way interaction is due to the presence of an interaction between condition and hemisphere at the frontal location, *F(2,44) = 6.31, p = .004, η*_*p*_^*2*^ *= 0.22*, but not at the parietal location, *F(2,44) = 0.04, p = .958, η*_*p*_^*2*^ *< 0.01*. At the frontal location, the different/new effect is bigger on the right hemisphere than on the left.

From 500 to 700 ms, the analysis identified a main effect of condition, *F(2,44) = 41.69, p < .001, η*_*p*_^*2*^ *= 0.66*, and interactions between condition and location, *F(1.5,33.32) = 4.92, p = .020, η*_*p*_^*2*^ *= 0.18*, and between condition, location and hemisphere, *F(2,44) = 5.76, p = .006, η*_*p*_^*2*^ *= 0.21*. All Bonferroni-corrected pair-wise comparisons were reliable (same/different $\overset{®}{x}$ = 1.72 ± 0.98, p < .001, d = 0.95; same/new $\overset{®}{x}$ = 3.70 ± 1.15, p < .001, d = 1.73; different/new $\overset{®}{x}$ = 1.98 ± 1.01, p < .001, d = 1.06). The interaction with location reflects more pronounced differences between the waveforms at the parietal location than at the frontal location. The three-way interaction is due to hemispheric differences at the frontal location that are not present at the parietal location (condition x hemisphere interactions: frontal location, *F(2,44) = 5.15, p = .010, η*_*p*_^*2*^ *= 0.19; parietal location*, *F(2,44) = 0. 13, p = .877, η*_*p*_^*2*^ *= 0.01*). At the frontal location, the size of the different/new contrast is bigger on the right hemisphere than on the left.

#### 3.3.2 Perceptual retrieval

**Fig. 7:**
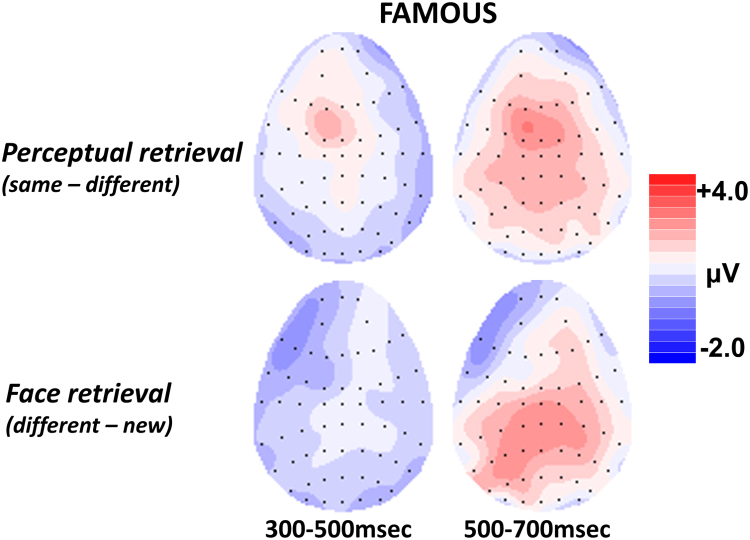
Topographic maps showing the distribution of ERP famous face effects. The different hit waveform has been subtracted from the same hit waveform to show the pattern of neural activity related to picture retrieval, and the new waveform has been subtracted from the different hit waveform to show neural activity associated with face retrieval. The pattern of neural activity observed for picture retrieval has a frontal distribution during both 300–500 and 500–700 msec latency periods. By contrast, neural activity observed for face retrieval has a left parietal distribution during the 500–700 msec latency period associated with ERP recollection effects.

From 500 to 700 ms, comparison of the same and different waveforms identified a main effect of condition, *F(1,22) = 20.79, p < .001, η*_*p*_^*2*^ *= 0.49*, but no interactions with electrode factors. Subsidiary analyses confirmed that this main effect is reliable at both the frontal, *F(1,22) = 13.42, p = .001, η*_*p*_^*2*^ *= 0.38*, and parietal, *F(1,22) = 20.01, p < .001, η*_*p*_^*2*^ *= 0.48*, locations. Fig. 7 shows the scalp topography of the perceptual retrieval and face retrieval effects.

#### 3.3.3 Face retrieval

From 500 to 700 ms, comparison of the different and new waveforms revealed a main effect of condition, *F(1,22) = 25.74, p < .001, η*_*p*_^*2*^ *= 0.54*, and an interaction between condition and location, *F(1,22) = 15.95, p = .001, η*_*p*_^*2*^ *= 0.42,* due to the difference between the waveforms being bigger at the parietal location, *F(1,22) = 38.73, p < .001, η*_*p*_^*2*^ *= 0.64,* than at the frontal location, *F(1,22) = 12.13, p = .002, η*_*p*_^*2*^ *= 0.36*.

### 3.4 Experiment 2 results summary

Experiment 2 used famous faces to examine the semantic status hypothesis of the functional significance of the anterior ERP old/new effect. Nie et al. (2014) advanced the hypothesis that the semantic status of an item determines whether it will produce a left parietal effect or an anterior effect. Experiment 2 contrasted ERPs for different picture hits and new faces to reveal neural activity associated with famous face retrieval. If the functional significance of the anterior effect is determined by semantic status, then famous face retrieval should have a left parietal maximum instead of the frontal maximum observed for unfamiliar faces in experiment 1. In experiment 2, a left parietal effect was observed for face retrieval, which provides partial support the semantic status hypothesis.

## 4 Comparing frontal and left parietal effects across participants

The face retrieval effects observed in different sets of subjects across experiments 1 and 2 were directly compared to assess whether the scalp distributions of the anterior and left parietal effects are dissociable. Evidence of topographic dissociation between the unfamiliar and familiar face effects would suggest that there are differences in the underlying neural populations generating the effects, consistent with the view that there are different cognitive operations involved in processing recognition memory as a function of an item's status in semantic memory.

**Fig. 8:**
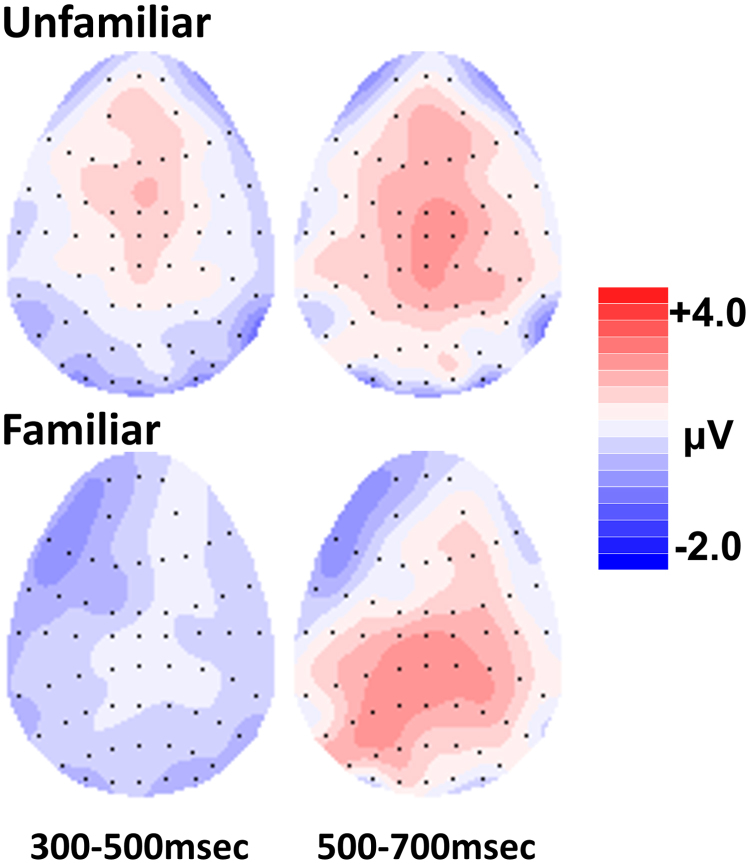
Topographic maps showing the distribution of ERP face retrieval effects for unfamiliar and famous faces. The new waveform has been subtracted from the different hit waveform to show neural activity associated with face retrieval. During the critical 500–700 msec latency period, a frontal effect is observed for unfamiliar faces while a left parietal effect is observed for famous faces.

Fig. 8 shows the contrast between the anterior ERP old/new effects (300–700 ms) observed for unfamiliar face recognition in experiment 1 and the left parietal effect (500–700 ms) observed for famous faces in experiment 2. Between-subjects analyses were performed to assess whether the unfamiliar face recognition effects could be topographically dissociated from the familiar face recognition effects. Rescaled difference waveforms (max-min method, McCarthy and Wood, 1985) were submitted to ANOVA with a between-subjects factor of semantic status (unfamiliar/famous) and within-subject factors of location (frontal/parietal) and hemisphere (left/right).

The first analysis was performed to assess whether the anterior effect observed for unfamiliar faces differs from the left parietal effect observed for famous faces from 500 to 700 ms. The analysis identified interactions between semantic status and location, *F(1,45) = 9.74, p = .003, η*_*p*_^*2*^ *= 0.18,* and between semantic status, location and hemisphere, *F(1,45) = 4.84, p = .033, η*_*p*_^*2*^ *= 0.*10. These topographic differences support the view that the anterior and left parietal effects observed for unfamiliar and famous faces are generated by partially non-overlapping neuronal populations, consistent with the view that dissociable retrieval processes support performance for each type of face.

A second analysis was performed to assess whether the anterior effect observed for unfamiliar faces from 500 m to 700 ms could be dissociated from the early frontal effect observed for famous faces in the 300–500msec latency period. The analysis revealed an interaction between semantic status, location and hemisphere, *F(1,45) = 4.75, p = .035, η*_*p*_^*2*^ *= 0.10*, consistent with the view that the two effects are produced by the activity of different underlying neuronal populations.

These between-subjects analyses comparing the scalp topography of the unfamiliar and famous effects therefore support the view that the anterior effect observed for unfamiliar faces at the frontal location from 500 m to 700 ms is dissociable from both the early frontal old/new effect and the left parietal effect observed for famous faces.

## 5 General discussion

This paper investigates how information about faces is retrieved from episodic memory. In particular, two experiments test competing accounts of the functional significance of a brain signal associated with recognition memory for visually complex stimuli such as faces. MacKenzie and Donaldson (2007) observed an anterior Event-Related Potential (ERP) old/new effect for unfamiliar faces; the component was modulated in a manner consistent with recollection and is observed during the same latency period as the left parietal old/new effect, which is widely understood to reflect processes linked with recollection. Experiment 1 investigated the *perceptual retrieval* hypothesis of the anterior effect (Galli and Otten, 2011), which argues that recollection of perceptual aspects of an episode is supported by a different process from recollection of other aspects of an episode. This hypothesis is derived from the observation of both anterior and parietal ERP old/new effects for recollection from 500 m to 700 ms. Galli and Otten linked the anterior effect with recollection of perceptual information. In the current paper, the perceptual retrieval account was investigated by contrasting ERPs for unfamiliar faces that were recognized from the same picture that was encoded or a different picture of an encoded face. Neural activity revealed by the contrast between same and different hit waveforms should in theory reflect perceptual retrieval, while the contrast between different hit and correct rejection waveforms reflects face retrieval *per se*. The anterior ERP old/new effect (500–700 ms) was observed for unfamiliar face retrieval but not for perceptual retrieval, which undermines the hypothesis advanced by Galli and Otten. Furthermore, the observation of an anterior effect for unfamiliar face retrieval, rather than a parietal effect, provides partial support for the alternative *status in semantic memory* hypothesis tested in experiment 2, which predicts that items not represented in long term memory pre-experimentally give rise to the anterior effect when recognized.

A second experiment using famous faces was performed to independently assess the *status in semantic memory* account (Nie et al., 2014) of the functional significance of the anterior effect. In experiment 2, a left parietal old/new effect was observed for famous face retrieval: there was no evidence of the anterior effect observed for unfamiliar faces in experiment 1. Crucially, a between-subjects topographic dissociation between the anterior effect observed for unfamiliar faces and the left parietal effect observed for famous faces provides support for the view that an item's status in semantic memory influences how it is recognized. The findings reported here demonstrate clearly that the presence or absence of semantic representations associated with an item determines which of the two retrieval processes associated with the anterior and parietal ERP old/new effects supports episodic memory.

Patterns of task behaviour were broadly similar across the two experiments. For both unfamiliar and famous faces, recognition memory was more accurate for same pictures than different pictures, which appears likely to be due to the match of perceptual information between study and test stimuli providing a boost to performance. In addition to perceptual retrieval enhancing recognition memory, semantic memory is well known to provide a boost to memory performance (Greve et al., 2007, La Corte et al., 2012). Across the experiments reported here, performance was superior for famous faces than for unfamiliar faces. This phenomenon might be explained by the presence of semantic representations stored in long term memory for the familiar faces, which would have facilitated encoding in a way that was unavailable for the unfamiliar faces, consistent with findings from the Reder lab (Reder et al., 2006, Reder et al., 2013). By this account, when presented with a famous face to encode, participants can make use of existing semantic structures to decrease cognitive load, such as committing a name or occupation to memory. For unfamiliar faces, meanwhile, for which no such semantic representations exist (except for gross categories such as sex, age, etc.), successful encoding involves the processing and retention of detailed visual information.

Reder et al. (2013) argue that unfamiliar faces cannot be recollected because encoding an unfamiliar stimulus uses up cognitive resources to such an extent that context cannot be bound to the episodic trace. From this perspective, successful encoding of an unfamiliar face can only lead to recognition memory supported by familiarity. While this model is internally coherent with respect to the R/K data collected in their manipulation of the frequency with which repeated background scenes served as context for facial stimuli, it does not accommodate reports of the anterior old/new effect functioning as if it provides information about recollection (MacKenzie and Donaldson, 2007, Galli and Otten, 2011). While one limitation of the current experiment is the absence of a process estimation procedure that could be used to assess the contributions of familiarity and recollection to recognition memory performance across conditions, the wider ERP literature is quite clear in providing support for the interpretation that famous and unfamiliar faces can be recollected in one of two different ways, and that the presence of absence of pre-existing semantic representations determines which recollection ERP effect is observed.

What these present findings cannot tell us directly is whether the faces in either experiment were recollected. Rather, interpretation of the processes supporting the effects observed for unfamiliar and famous face retrieval rest upon previous literature, which has linked the left parietal effect unambiguously with recollection (Rugg and Yonelinas, 2003, Rugg and Curran, 2007). The literature showing that the anterior effect reflects recollection is smaller, yet convincing (MacKenzie and Donaldson, 2007, MacKenzie and Donaldson, 2009, Galli and Otten, 2011). If we assume that the faces in the present experiments were recollected, and consider the clear support for semantic status in determining which brain signal is observed, then what can we infer about the processing of recollection? One possibility is that there is a common core retrieval process which acts upon information differently depending on whether or not it is semantically represented. From this perspective, one processing route might involve hippocampal projections to the neocortex, where stimulus-specific representations have been consolidated, while another processing route may involve hippocampal projections to visual association areas, for example, where recently encoded perceptual information might be represented. An alternative to this model could be two processes that are entirely separate from one another, potentially engaging distinct medial temporal lobe regions in addition to different cortical structures. Kafkas et al. (2017) have found no evidence of material specificity for hippocampal recollection in a study using objects, scenes and faces as stimuli. On this basis, it would appear that the former possibility of common hippocampal activity projecting to distinct cortical regions is more likely. However, all that can be concluded on the basis of the electrophysiological evidence presented here is that there is a difference somewhere along the processing chain in recognition memory of information depending upon whether it is represented in semantic memory or not. Further work is required to understand the processes involved in semantic consolidation, and to investigate the boundary conditions between the engagement of the processes producing the anterior and left parietal ERP old/new effects.

Another issue raised by the present study is the nature of the processes that support perceptual retrieval. No strong conclusions can be drawn from experiment 1 using unfamiliar faces, since the posterior effect observed in the contrast between same and different hit waveforms was only discovered by focused analysis of parietal electrodes. A more robust effect might have come to light in the omnibus ANOVA. Nevertheless, a weak effect with a posterior scalp topography was observed during the 500–700 ms latency period. The perceptual retrieval effect observed for famous faces in experiment 2 had a larger effect size at the parietal location than the frontal location. The timing and distributions of these effects are difficult to interpret definitively; however, one possibility is that the perceptual retrieval effects reflect delayed familiarity assessment. In support of this idea, posterior old/new effects have been observed for familiarity previously (MacKenzie and Donaldson, 2007, Bridger et al., 2014), although most studies using lexical stimuli interpret the midfrontal old/new effect (300–500 ms), or FN400, as a brain signal linked with familiarity. As such, if the posterior perceptual retrieval effects observed here reflect familiarity then familiarity must be just as susceptible to fractionation as recollection.

In summary, different patterns of brain activity can be observed for recognition memory for unfamiliar and famous faces, whose status in semantic memory varies. Unfamiliar face retrieval is associated with an anterior ERP old/new effect, whereas famous face retrieval is associated with a topographically dissociable left parietal effect. These results support the view that an item's status in semantic memory determines how it is retrieved from episodic memory, advanced by Nie et al. (2014), and fail to support the competing view that the anterior effect reflects retrieval of perceptual aspects of an episode (Galli and Otten, 2011). The way that humans remember an old friend's face appears to be different from the way that somebody one has only met once is remembered. The same functional outcome, the recognition of episodic information, is thus achieved via dissociable neural pathways.

## Footnotes

1 Although the functional significance of the FN400 remains contested (Hou et al., 2013, Wang et al., 2015), the association between the left parietal effect and recollection-related processing is widely accepted.
